# Immersive Media Presentation and Preschoolers’ Prosocial Behaviors: The Mediating Role of Theory of Mind

**DOI:** 10.3389/fpsyg.2022.889475

**Published:** 2022-06-03

**Authors:** Ting Chen, Chenglin Jin

**Affiliations:** ^1^School of Humanities and Communication, Anhui Xinhua University, Hefei, China; ^2^School of Business, Anhui Xinhua University, Hefei, China; ^3^School of Management, University of Science and Technology of China, Hefei, China

**Keywords:** prosocial behaviors, immersive media presentation, traditional media presentation, theory of mind, empirical research

## Abstract

Although scholars have asserted that it is necessary to explore the effect of immersive media presentation on preschoolers’ prosocial behaviors, the empirical research, as well as the moderating roles of this link, remained limited. One hundred and twenty preschoolers (mean age = 5.4 years) were involved in three experiments of four waves. This study empirically examined the effect of media presentation, including immersive media presentation and traditional media presentation, on preschoolers’ prosocial behaviors and the moderating effect of theory of mind (ToM) on such links. After the experimental intervention, we find that the extent to which traditional media presentation and immersive media presentation impact preschoolers’ prosocial behaviors is different. First, the results show that preschoolers, who have been involved in traditional media presentation, do not notably reveal the development of their prosocial behaviors. However, in the group of high ToM, we find that traditional media presentation positively and significantly relates to preschoolers’ prosocial behaviors. That is to say, the positive influence of traditional media presentation depends on ToM. Second, the results also show that immersive media presentation positively and significantly relates to preschoolers’ prosocial behaviors whether ToM is high or not. Furthermore, ToM encourages the positive influence of immersive media presentation on preschoolers’ prosocial behaviors.

## Introduction

Prosocial behaviors, the voluntary physical reactions intending to benefit others (Dunfield and Kuhlmeier, 2013; Yu et al., 2020), are widely regarded as a critical topic in developmental psychology and educational psychology (Newton et al., 2014; Paulus, 2014; Imuta et al., 2016; Benish-Weisman et al., 2019; Wu et al., 2020; Zeng et al., 2020). Guidelines for Kindergarten Education, issued by the Ministry of Education of China in 2001, puts forward four goals about the development of preschoolers’ prosocial behaviors, such as the willingness to comfort, cooperate, help, and share (Dunfield and Kuhlmeier, 2013). This policy points out the importance of the research on preschoolers’ prosocial behaviors. In addition, prior studies indicated that preschoolers’ prosocial behaviors positively relate to their future development (Lohndorf et al., 2019), interpersonal harmony (Zeng et al., 2020), and social implications (Malonda et al., 2019). Therefore, it is essential to explore how to promote preschoolers’ prosocial behaviors (Yu et al., 2020).

Prior studies indicated that media presentation may impact the development of preschoolers’ prosocial behaviors. For example, Chang et al. (2011) indicated that traditional media presentation has potential influence on preschoolers’ prosocial behaviors (Franklin, 2004), because media technology enables the information conveyance and storage (Sanchez and Fairfield, 2003). The study of Flook et al. (2015) also contended that the curriculum with a mindfulness-based kindness can promote preschool children’s prosocial behaviors, such as the behaviors of helping and learning from each other. With the development of immersive media technologies (i.e., Virtual Reality, 360° Imagery or Video, and 3D Content), scholars in domain of developmental psychology and educational psychology called for more grainy research about the relationship between the use of immersive media technologies and preschoolers’ development. For example, Widowati (2018) documented that the alignment of watching animations and role-play games in immersive virtual environments will generate a better effect on learning outcomes. Ma (2020) proposed that immersive stories have a positive influence on students’ prosocial attitudes and willingness to help. Although prior scholars noted that the use of advanced immersive technique plays a critical role in enhancing preschoolers’ helping and sharing behaviors (Ferguson, 2015; Benish-Weisman et al., 2019), the empirical investigation on the effect of immersive media presentation on preschoolers’ prosocial behaviors still remains limited. In this study, we differentiate the effect of immersive media presentation on preschoolers’ prosocial behaviors from the traditional media presentation to response to scholarly calls and propose our first research question as follow:

*RQ1*: How immersive media presentation and traditional media presentation affect preschoolers’ prosocial behaviors? And what are the differences?

Additionally, previous research indicated that the degree to which media presentation impacts students’ behaviors depends on their cognitive capability (Imuta et al., 2016), such as the theory of mind (ToM). ToM refers to an individual cognitive ability to understand psychical states. Such cognitive ability can facilitate preschoolers to understand others’ affection and intention, as well as to predict and interpret others’ behaviors (Wellman et al., 2001; Lecce et al., 2014; Bigelow et al., 2021). ToM was widely regarded as a critical enabler of social living and social reasoning (Meins et al., 2002; Peterson and Wellman, 2019). Prior study revealed that preschoolers’ ability to understand others’ feelings and behaviors may improve the effect of teaching approaches on preschoolers’ learning progress (Imuta et al., 2016). In this logic, we contend that for preschool children, ToM may encourage the effect of media presentation on their prosocial behaviors. That is to say: ToM, a typical cognitive capability, may play a critical moderating effect on the relationship between media presentation (i.e., immersive media presentation and traditional media presentation) and preschoolers’ prosocial behaviors. However, few empirical studies have investigated the moderating role of ToM in this link. Thus, we propose another research question:

*RQ2*: How ToM moderates the relationship between the media presentation (i.e., immersive media presentation and traditional media presentation) and preschoolers’ prosocial behaviors?

To untangle the two research questions, we used the data deriving from our three experiments of four waves to empirically examine the effect of media presentation (i.e., immersive media presentation and traditional media presentation) on preschoolers’ prosocial behaviors and the moderating role of ToM in such link. We make two key contributions in this study. First, we enrich the research about the relationship between immersive media and preschoolers’ prosocial behaviors through empirically exploring how immersive media presentation impacts preschoolers’ prosocial behaviors, and differentiating that from the effect of traditional media presentation on preschoolers’ prosocial behaviors. Second, we also expand the contingencies of the relationship between media presentation, including immersive media presentation and traditional media presentation, and preschoolers’ prosocial behaviors through empirically investigating the moderating effect of ToM. We thus respond to the scholarly call for more research on preschoolers’ prosocial behaviors (Lockwood et al., 2014; Lohndorf et al., 2019). In our study, we find that after the experimental intervention, the extent to which traditional media presentation and immersive media presentation impact preschoolers’ prosocial behaviors is different. First, the results show that preschoolers who have been involved in traditional media presentation do not notably reveal the development of their prosocial behaviors. However, in the group of high ToM, we find that traditional media presentation positively and significantly relates to preschoolers’ prosocial behaviors. That is to say, the positive influence of traditional media presentation depends on ToM. Second, the results also show that immersive media presentation positively and significantly relates to preschoolers’ prosocial behaviors whether ToM is high or not. Furthermore, ToM encourages the positive influence of immersive media presentation on preschoolers’ prosocial behaviors. Our findings thus provide the teachers or managers of kindergarten with underlying approaches to facilitate preschoolers’ prosocial behaviors.

## Hypothesis Development

Prosocial behaviors refer to the voluntary physical reactions intending to benefit others (Dunfield and Kuhlmeier, 2013; Yu et al., 2020). Prior studies showed several antecedents of preschoolers’ prosocial behaviors, such as parents’ discourse about emotions with their young children (Brownell et al., 2013), parents’ values (i.e., self-enhancement values, self-transcendence value, and self-conservation values; Benish-Weisman et al., 2019), peer influence and status (Choukas-Bradley et al., 2015), interpersonal synchrony (Cirelli et al., 2014), moral perfectionism (Zeng et al., 2020), and parental sensitivity (Newton et al., 2014). Besides these factors, prior scholars also indicated that appropriate training and education can enhance the preschoolers’ intentions of prosocial behaviors. For example, Ferguson (2015) noted that children’s prosocial behaviors can be guided when they are watching video-games. Flook et al. (2015) and Hafenbrack et al. (2020) reported that the mindfulness-based kindness courses promotes preschool children’s prosocial behaviors. However, few researches have empirically explored the effect of media presentation, especially immersive media presentation, on preschoolers’ prosocial behaviors.

Immersive media presentation refers to a teaching presentation involved in immersive virtual technologies (Ma, 2020), while traditional media presentation reflects a teaching presentation synchronized with images, words, audios, and videos (Yetlen and Nunamaker, 1991; Patrick, 2015; Widowati, 2018). Children’s prosocial orientation can be promoted when they consciously copy the behaviors from the videos or cartoons that they have watched whether using traditional media technologies or immersive media technologies (Carpenter et al., 2013). Watching videos or cartoons using different media technologies may have distinct effect on facilitating the tendency of preschoolers’ prosocial behaviors (Helt et al., 2020). Immersive media, such as virtual reality, augmented reality, and 360° video, offers preschoolers so high a degree of vividness that they can better understand what have been conveyed. In addition, immersive media technologies can also help to reduce the cognitive and psychological difficulties through amplifying the feeling of spatial presence; thus, compared with traditional media presentation, watching cartoons embedded with immersive media technologies have a stronger positive influence on children’s tendency to mimic what they have learned (Widowati, 2018). Following these logic, we contend that the effect of immersive media presentation on preschoolers’ prosocial behaviors is stronger, compared with the impact of traditional media presentation. Based on the argument above, we propose three hypotheses as follows:

*Hypothesis 1*: Both immersive media presentation and traditional media presentation related positively to the development of preschoolers’ prosocial behaviors. Furthermore, the effect of immersive media presentation on preschoolers’ prosocial behaviors is stronger than that of traditional media presentation.

Prior scholars noted that preschoolers’ development of ToM related positively to their executive functioning (Carlson et al., 2002, 2004; Sabbagh et al., 2006), children’s language development (Astington and Jenkins, 1999; Ruffman et al., 2002; Milligan et al., 2007), moral judgments (Gonultas et al., 2021). For example, Kim et al. (2021) have noted that ToM skills relate to discourse understanding and the relationship between ToM and narrative understanding is more stronger than the relationship between ToM and informational text understanding. Prior psychological scholars also indicated that the social behaviors acceptable results from the continuous interactions of social affective and social cognitive processes, considering these processes are executed by separable and independent area of brains (Preckel et al., 2018). In addition, children’s theory of mind plays an essential positive role when they reading and understanding others’ feelings and behaviors (Kidd and Castano, 2013). Follow this logic, we propose the hypothesis as follow:

*Hypothesis 2*: Preschoolers’ ToM ability strengthens the positive influence of media presentation (including traditional media presentation and immersive media presentation) on their development of prosocial behaviors.

## Methodology

In our study, we focus on the preschoolers located in the China’s Yangzi River Delta. First, before the preschoolers attended the training courses, we measured their original prosocial behaviors through watching them. Second, the preschoolers chosen would be arranged to watch the teaching materials prepared in advance. We then watched and recorded their change in such behaviors. Third, after experimentation, we would compare their behavior between before and after experimentations.

### Participants

Two hundred and eighteen preschoolers were randomly selected from several kindergartens located in Yangtze River Delta of China. Before the formal experiment, we conducted an experiment regarding preschoolers’ prosocial behaviors, and 45 % of these preschoolers showed such behaviors. Then, we choose the rest, 120 preschoolers, who failed the prior test as our research sample and divided them into two groups. One group was arranged to participate in the courses with immersive virtual technologies to investigate the effect of immersive media presentation on preschoolers’ prosocial behaviors, and the other group was settled to take part in the courses with traditional media technologies to explore the effect of traditional media presentation on preschoolers’ prosocial behaviors.

### Measures

#### Prosocial Behaviors

We adapted the research of Newton et al. (2014) and Lohndorf et al. (2019) to measure the development level of preschoolers’ prosocial behavior. We first set up two hypothetical scenarios for every category of prosocial behaviors, including the behaviors of helping, comforting, sharing, and cooperating, added up to 8 scenarios. Then, we observed and recorded their behaviors. Two points would be scored when children have the spontaneous prosocial behaviors. One point would be scored if they showed the prosocial behaviors only with the experimenters’ linguistic hints. Otherwise, we would mark this item as zero point. The aggregate scores of the eight items revealed the level of prosocial behavior development, ranging from 0 to 16. Furthermore, in order to avoid common method bias, we differentiated the hypothetical scenarios in post-test from those in pre-test. Similarly, all the selected scenarios are consistent with the level of preschoolers’ cognitive development, and the scoring criteria is the same.

#### Theory of Mind

We adapted the research of Melot and Angeard (2003) and Misailidi and Kapsali (2020) to measure the ToM. This measurement included five items covering the test of unexpected-location tasks (two items), unexpected-content tasks (two items), and appearance-reality task (one item). If the subjects respond to each item correctly, they would be scored one point. The aggregate scores of the five items are five points.

#### Media Presentation

We first adopted the teaching methods of traditional media presentation. Eight clips from domestic animations with scenarios for helping, cooperating, sharing, and comforting were selected and evaluated by several scholars who specializes in prosocial behaviors. We then aligned these eight animation clips into teaching schemes. Forty subjects were arranged to watch two animation clips every week. The whole experiment lasted for 4 weeks. We also adopted the teaching methods of immersive media presentation. The subjects wearing head-mounted displays watched eight animation clips and their subsequent behaviors were evaluated by the scholars who specializes in preschoolers’ prosocial behaviors. The theme of these animation clips is also related to the prosocial behaviors, such as the behaviors of helping, cooperating, comforting, and sharing. This experiment also lasted for 4 weeks.

### Research Procedure

#### The Pre-test of Preschoolers’ Prosocial Behaviors

The research process of our study is divided into four waves. In the first wave, based on the sample pool of two hundred and eighteen subjects, we conducted a pre-test in which our research assistants were arranged to observe whether the chosen preschoolers showed the tendency of prosocial behaviors, such as the ones to help or comfort others. According to the alignment of our on-site observations and subsequent analyses, we labeled the ones who have not revealed the prosocial behaviors. The whole pre-test lasted 5 days and we got 120 samples for subsequent experiments.

#### The Experimentation of Theory of Mind

In the second wave, after the pre-test, based on 120 preschoolers who failed the pre-test, we conducted another experimentation about ToM which used to examine the cognitive capability to understand others’ emotions and behaviors. In such experimentation, we collected and recorded the data about preschoolers’ capability to understand others’ feeling and behaviors. According to the scores of this experimentation, we divided the subjects into two parts, namely high ToM and low ToM. The data were used to analyze the contingent effect of ToM on the relationship between media presentation (including traditional media presentation and immersive media presentation) and preschoolers’ prosocial behaviors. This experimentation lasted 2 days.

#### The Experimentation of Media Presentation

In the third wave, the 120 preschoolers who have not showed prosocial behaviors in the pre-test were randomly divided equally into three groups. First, for the 40 preschoolers in group one, we conducted an experimentation on traditional media presentations. This experimentation would last 4 weeks, in which the chosen preschoolers would be involved in watching eight animation clips. During the process, our research assistant would watch and record the preschoolers’ behaviors. Second, at the same time, based on another 40 subjects in group two, we simultaneously conducted the experimentation on immersive media presentation lasting 4 weeks. In this 4 weeks, the chosen preschoolers were involved in watching eight animation clips with the elements of prosocial behaviors through wearing head-mounted displays. Our research assistants are also required to observe and document the subjects’ actions. Third, the rest 40 preschoolers were arranged into the controlling group that would not be involved in the experimentation of media presentation.

#### The Post-test of Preschoolers’ Prosocial Behaviors

In the fourth wave, after the experiments of ToM and media presentation, based on the 120 subjects, we subsequently conducted a post-test of prosocial behaviors. To alleviate the common method bias, we adopted another measurement in the post-text. The standards and procedures in the post-test are the same with those in the pre-test. This test lasted for 2 days.

## Results

### Media Presentation and Preschoolers’ Prosocial Behaviors

Having finished all experiments, we applied SPSS 26.0 software to process and analyze the data collected in four waves (Jin et al., 2022). We anticipated that the preschoolers who were involved in the experimentation of media presentation, including traditional media presentation and immersive media presentation, are more likely to show the prosocial behaviors than those who have not.

Specifically, in terms of the relationship between the behaviors of comforting others and media presentation, the result of covariance analysis shows that the effect of immersive media presentation and traditional media presentation on preschoolers’ behaviors of comforting is different (*F* = 11.087, *p* < 0.001). We further conducted a *post hoc* examination to verify the results. The results show that after the intervention, the scores of the group who have taken the courses with immersive media presentation are 0.869(*p* < 0.001) higher than the scores of controlling group and are 0.656 (*p* < 0.05) higher than that of the group who have taken the courses with traditional media presentation, while the score difference between the group who have taken the courses with traditional media presentation and the controlling group is insignificant.

In terms of the relationship between the behaviors of cooperating others and media presentation, the result of covariance analysis shows that the effect of immersive media presentation and traditional media presentation on preschoolers’ behaviors of comforting is different (*F* = 12.543, *p* < 0.001). We further conducted a *post hoc* examination to verify the results. The results show that after the intervention, the scores of the group who have taken the courses with immersive media presentation are 0.860 (*p* < 0.001) higher than the scores of controlling group and are 0.600 (*p* < 0.05) higher than that of the group who have taken the courses with traditional media presentation, while the score difference between the group who have taken the courses with traditional media presentation and the controlling group is also insignificant.

In terms of the relationship between the behaviors of helping others and media presentation, the result of covariance analysis shows that the effect of immersive media presentation and traditional media presentation on preschoolers’ behaviors of comforting is different (*F* = 21.602 *p* < 0.001). We further conducted a *post hoc* examination to verify the results. The results show that after the intervention, the scores of the group who have taken the courses with immersive media presentation are 1.082 (*p* < 0.001) higher than the scores of controlling group and are 0.692 (*p* < 0.001) higher than that of the group who have taken the courses with traditional media presentation. Meanwhile, the scores of the group who have taken the courses with traditional media presentation are 0.390 (*p* < 0.05) higher than the scores of the controlling group.

In terms of the relationship between the behaviors of sharing with others and media presentation, the result of covariance analysis shows that the effect of immersive media presentation and traditional media presentation on preschoolers’ behaviors of comforting is different (*F* = 30.176 *p* < 0.001). We further conducted a *post hoc* examination to verify the results. The results show that after the intervention, the scores of the group who have taken the courses with immersive media presentation is 1.095 (*P* < 0.001) higher than the scores of controlling group and is 0.430 (*p* < 0.05) higher than that of the group who have taken the courses with traditional media presentation. Meanwhile, the scores of the group who have taken the courses with traditional media presentation are 0.665 (*P* < 0.001) higher than the scores of the controlling group.

In H1, we predict that both immersive media presentation and traditional media presentation positively related to preschoolers’ prosocial behaviors and that the effect of immersive animation presentation on prosocial behaviors is stronger than that of traditional media presentation. According to research results mentioned above, although immersive media presentation can both promote four types of prosocial behavioral development, traditional media presentation only facilitates the helping and sharing behaviors. Furthermore, the effect of immersive media presentation on preschoolers’ prosocial behaviors (e.g., the comforting, cooperating, helping and sharing behaviors) is stronger than that of traditional media presentation. Thus, H1 is partly supported (see Table 1).

**Table 1:** Results of the relationship between media presentation and prosocial behaviors.

		Immersive media presentation	Traditional media presentation	Controlling group
		Model 1	Model 2	Model 3
Comforting	Pre-test	1.60 ± 1.74	1.47 ± 0.60	1.62 ± 0.67
	Post-test	2.53 ± 1.18	1.78 ± 0.92	1.67 ± 0.86
Cooperating	Pre-test	1.78 ± 0.80	1.65 ± 0.66	1.48 ± 0.64
	Post-test	2.60 ± 1.03	1.90 ± 0.87	1.50 ± 0.96
Helping	Pre-test	1.13 ± 0.72	1.15 ± 0.70	1.35 ± 0.66
	Post-test	2.30 ± 0.97	1.63 ± 0.87	1.38 ± 0.81
Sharing	Pre-test	1.73 ± 1.01	1.70 ± 0.94	1.63 ± 0.77
	Post-test	2.75 ± 1.06	2.30 ± 0.99	1.57 ± 0.84

### The Moderating Role of ToM

In the second wave, according to the scores of ToM, the subjects were labeled as high ToM (ToM_H) or low (ToM_L). The results of covariance analysis show that the extent to which media presentation impacts preschoolers’ prosocial behaviors depends on the role of ToM. After the intervention of media presentation, the preschoolers with high ToM show an even stronger tendency of prosocial behaviors. Specifically, in terms of the effect of media presentation on the behaviors of comforting, when ToM is higher, after the intervention of immersive media presentation, preschoolers are more likely to show the behaviors of comforting (*F* = 16.975, *p* < 0.0*5*) comparing with the intervention of traditional media presentation (see Table 2). Considering the effect of media presentation on the behaviors of cooperating, when ToM is higher, after the intervention of immersive media presentation, preschoolers are more likely to show the behaviors of cooperating (*F* = 11.79, *p* < 0.05, *η*^2^ = 0.169) comparing with the intervention of traditional media presentation (see Table 2). Considering the effect of media presentation on the behaviors of helping, when ToM is higher, after the intervention of immersive media presentation, preschoolers are more likely to show the behaviors of helping (*F* = 14.026, *p* < 0.05, *η*^2^ = 0.195) comparing with the intervention of traditional media presentation (see Table 2). Considering the effect of media presentation on the behaviors of sharing, when ToM is higher, after the intervention of immersive media presentation, preschoolers are more likely to show the behaviors of sharing (*F* = 19.414 *p* < 0.05, *η*^2^ = 0.251) comparing with the intervention of traditional media presentation (see Table 2). Thus, consistent with our expectation, the role of ToM encourages the positive effect of media presentation, including immersive media presentation and traditional media presentation, on preschoolers’ prosocial behaviors; thus, H2 is supported.

**Table 2:** Results of moderating effect of ToM in the relationship between media presentation and prosocial behaviors.

		Immersive media presentation		Traditional media presentation		Controlling group	
		Model 1 ToM_H	Model 2 ToM_L	Model 3 ToM_H	Model 4 ToM_L	Model 5 ToM_H	Model 6 ToM_L
Comforting	Pre-test	1.65 ± 0.81	1.55 ± 0.69	1.56 ± 0.73	1.42 ± 0.50	1.58 ± 0.84	1.67 ± 0.48
	Post-test	3.30 ± 0.73	1.75 ± 1.02	2.50 ± 0.63	1.29 ± 0.75	1.89 ± 0.94	1.48 ± 0.75
Cooperating	Pre-test	1.80 ± 0.77	1.75 ± 0.85	1.88 ± 0.72	1.50 ± 0.59	1.58 ± 0.51	1.38 ± 0.74
	Post-test	3.30 ± 0.80	1.90 ± 0.72	2.38 ± 0.72	1.58 ± 0.83	1.79 ± 0.71	1.24 ± 2.09
Helping	Pre-test	1.25 ± 0.72	1.00 ± 0.73	1.06 ± 0.68	1.21 ± 0.72	1.21 ± 0.71	1.48 ± 0.60
	Post-test	2.40 ± 1.10	2.20 ± 0.83	2.25 ± 0.77	1.21 ± 0.66	1.32 ± 1.00	1.43 ± 0.60
Sharing	Pre-test	1.75 ± 1.07	1.70 ± 0.98	1.94 ± 0.85	1.54 ± 0.98	1.84 ± 0.76	1.43 ± 0.75
	Post-test	3.00 ± 1.12	2.50 ± 0.95	2.81 ± 0.83	1.96 ± 0.95	2.05 ± 0.78	1.14 ± 0.65

## Discussion

Our experimentation results reveal that those preschoolers who have participated in the courses with the immersive media presentation are more likely to show their prosocial behaviors, such as the ones to comfort or help others and the ones to share or cooperate with others. That is to say, the teaching approaches of media presentation using immersive virtual devices enable preschoolers’ prosocial behaviors. Furthermore, we also find that the immersive media presentation has a stronger effect on preschoolers’ prosocial behaviors than that of traditional media presentation. A possible explanation is that immersive media (e.g., 360° imagery or video, virtual reality, and 3D content) can make the teaching materials more comprehensible and be used to reduce the psychological and cognitive gap. Thus, this approach is more advisable.

Our study also explores the moderating role of ToM in the relationship between different approaches of media presentation and preschoolers’ prosocial behaviors. The experimentation results reveal that both immersive media presentation and traditional media presentation have a stronger influence on preschoolers’ prosocial behaviors when their ability to sense and understand others’ emotions and behaviors is higher. That is to say, the ToM has a positive contingent effect on the relationship between media presentation and preschoolers’ prosocial behaviors. A possible explanation is that the external knowledge or information need to be processed by internal cognitive capability, especially for preschoolers whose ability to understand others behaviors varies with each individual.

### Theoretical Implication

In our study, we integrate the role of media presentation and ToM to explore how to facilitate preschoolers’ prosocial behaviors. Our research team makes two theoretical contributions. First, we enrich the understanding of how to guide preschooler’s prosocial behaviors based on teaching methods using immersive media technologies and traditional media technologies. Scholars in education management called for more empirical investigation about the role of immersive media in promoting preschoolers’ prosocial behaviors. Focusing on media presentation, we empirically explore the effect of immersive media presentation and traditional media presentation on preschoolers’ prosocial behaviors, which respond in part to the scholarly call. In addition, we contribute to the studies of contingent factor in the relationship between media presentation and preschoolers’ prosocial behaviors. We find that the extent to which the media presentation impacts preschoolers’ prosocial behaviors relies on preschoolers’ capability to understand others’ behaviors (Wellman et al., 2001). Our research confirms the necessity to realize the importance of training preschoolers’ cognitive abilities while improving teaching approaches. We thus also extend the understandings of the contingent conditions of the research of preschoolers’ prosocial behaviors.

### Practical Implication

Our study supplies three primary implications with parents, kindergartens, and the society. First, for kindergartens and preschoolers’ parents, they should deepen the understanding that preschoolers’ prosocial behaviors play a critical role in promoting their healthy development and these behaviors can be trained through taking appropriate teaching courses. Furthermore, comparing with the teaching method using traditional media technologies, using immersive media technologies will generate a better positive effect. Thus, both kindergartens and families should highlight the daily collection of ideal teaching materials with the elements of prosocial behaviors, especially those cartoon materials. Second, considering the teaching materials, such as the animations and cartoons with the elements of prosocial behaviors, can direct preschoolers’ behaviors effectively, and because of the relative deficiency in the development of domestic cartoons compared with several developed countries, it is essential for our society to encourage the creation and distribution of superior domestic animations. Furthermore, the details of the cartoons should be under the strict supervision (Fedorov et al., 2018). Third, our study also finds that the extent to which media presentation affect preschoolers’ prosocial behaviors depends on the theory of mind. Preschoolers’ capability to sense and understand others’ feelings and behaviors plays a critical role in the positive effect of media presentation on their prosocial behaviors. Thus, to guide the preschoolers’ behaviors in a greater extent, all the teaching units, including kindergartens, families, and the society, should not only focus on taking the immersive-media-embedded and traditional-media-embedded teaching measures but also should emphasize on enhancing preschoolers’ capability to sense and understand others’ feelings and behaviors at the same time.

### Limitations and Future Research

Three limitations suggest directions for future research. First, the samples of our study are confined to kindergartens in China. Thereby, it may be risky to generalize our research conclusions to other countries and regions. Future research covering more regions may enrich our research. Second, although our experimentations are consisted of the data of four waves, the sample size remains to be enlarged. Efforts to enlarge the size of samples, as well as the size of research team, may be extend our findings. Third, our research team only explores the contingent roles of preschoolers’ capability to understand others’ feelings and behaviors. Future research may contribute to our study by investing the potential moderating influence of social and environmental factors on the relationship between media presentation and preschoolers’ prosocial behaviors.

## Data Availability Statement

The raw data supporting the conclusions of this article will be made available by the authors.

## Author Contributions

TC involved in conceptualization, methodology, and writing—original draft preparation. CLJ took part in methodology, writing—reviewing and editing, and funding acquisition. All authors have read and agreed to the submitted version of the manuscript.

## Funding

This study was supported by Education Department of Anhui Province (grant numbers gxbiZD2021018 and gxyq2021046), Anhui Xinhua University (grant numbers 2019rw013 and 2021jy005), and Department of Hefei Science and Technology (grant number 2020005).

## Conflict of Interest

The authors declare that the research was conducted in the absence of any commercial or financial relationships that could be construed as a potential conflict of interest.

The Reviewer J-BL declared a shared affiliation with the authors to the handling editor at the time of review.

## Publisher’s Note

All claims expressed in this article are solely those of the authors and do not necessarily represent those of their affiliated organizations, or those of the publisher, the editors and the reviewers. Any product that may be evaluated in this article, or claim that may be made by its manufacturer, is not guaranteed or endorsed by the publisher.
